# RF Remote Blood Glucose Sensor and a Microfluidic Vascular Phantom for Sensor Validation

**DOI:** 10.3390/bios11120494

**Published:** 2021-12-03

**Authors:** Muhammad Farhan Affendi Mohamad Yunos, Rémi Manczak, Cyril Guines, Ahmad Fairuzabadi Mohd Mansor, Wing Cheung Mak, Sheroz Khan, Noor Amalina Ramli, Arnaud Pothier, Anis Nurashikin Nordin

**Affiliations:** 1 Department of Electrical and Computer Engineering, Kulliyyah of Engineering, International Islamic University Malaysia, Kuala Lumpur 53100, Malaysia; farhanfendi93@gmail.com (M.F.A.M.Y.); fairuzabadimansor@gmail.com (A.F.M.M.); amalina_bella@yahoo.com (N.A.R.); 2 XLIM-UMR 7252, University of Limoges/CNRS, 87060 Limoges, France; Remi.MANCZAK@chu-limoges.fr (R.M.); Cyril.guines@unilim.fr (C.G.); arnaud.pothier@xlim.fr (A.P.); 3 Biosensors and Bioelectronics Centre, Department of Physics, Chemistry and Biology (IFM), Linköping University, 58183 Linkoping, Sweden; wing.cheung.mak@liu.se; 4 Manager Department of Electrical Electronics and Renewable Engineering, Onaizah Colleges of Engineering, P.O. Box 2053, Unayzah 56453, Saudi Arabia; cnar32.sheroz@gmail.com

**Keywords:** blood glucose monitoring, diabetes, non-invasive sensor, PDMS, vascular phantom, glucometer, RF sensor

## Abstract

Diabetes has become a major health problem in society. Invasive glucometers, although precise, only provide discrete measurements at specific times and are unsuitable for long-term monitoring due to the injuries caused on skin and the prohibitive cost of disposables. Remote, continuous, self-monitoring of blood sugar levels allows for active and better management of diabetics. In this work, we present a radio frequency (RF) sensor based on a stepped impedance resonator for remote blood glucose monitoring. When placed on top of a human hand, this RF interdigital sensor allows detection of variation in blood sugar levels by monitoring the changes in the dielectric constant of the material underneath. The designed stepped impedance resonator operates at 3.528 GHz with a Q factor of 1455. A microfluidic device structure that imitates the blood veins in the human hand was fabricated in PDMS to validate that the sensor can measure changes in glucose concentrations. To test the RF sensor, glucose solutions with concentrations ranging from 0 to 240 mg/dL were injected into the fluidic channels and placed underneath the RF sensor. The shifts in the resonance frequencies of the RF sensor were measured using a network analyzer via its S_11_ parameters. Based on the change in resonance frequencies, the sensitivity of the biosensor was found to be 264.2 kHz/mg·dL^−1^ and its LOD was calculated to be 29.89 mg/dL.

## 1. Introduction

Diabetes is a chronic disease that is on the rise across the globe, especially amongst developed nations. To date, there is no known way to prevent or cure diabetes; however, the patient’s quality of life can be improved by having periodic monitoring and quantification of glucose levels. Continuous self-monitoring of blood glucose levels allows patients to better control their diet and medication intake, leading to more stable blood sugar levels and fewer incidents of hypo and hyperglycemia [1]. The early generations of glucose sensors were chemical-based, a manual operation mostly involving blood sampling [2]. With the advance of technology and complexity of devices, the manual sampling of blood glucose has become outdated and should be improved for better monitoring of patients. Microwave sensing using small, printed high-frequency sensors is one of the promising techniques to implement non-invasive glucose monitoring. The general principle is to utilize electromagnetic waves to characterize the material under test (MUT), based on its dielectric properties and measuring its resonant frequencies. Previous studies have found that these resonant frequencies correlate to dielectric permittivity of the material and shift with varying concentrations of glucose [3]. Permittivity values are inversely proportional to glucose concentration, where higher concentrations result in lower permittivity values. Different designs of radio frequency (RF) resonator sensors can be used to detect permittivity changes in a material such as circular [4], interdigital transducers (IDT) [5], near-field [6] and microstrip [7]. IDT is the most common design for a wide range of applications such as chemical sensor [8], food inspection [9] and humidity sensor [10,11]. IDT structure has also been used as a biosensor where it was used for direct, rapid quantification and detection of prostate-specific antigens [12]. To enhance the performance of the IDT sensor, stepped impedance structures can be added to the IDT design to improve the quality factor and impedance values of the resonator. These designs, known as stepped impedance resonators (SIR), are compact, have small footprints and allow independent control of the characteristic impedance of the resonator [13]. The RF sensors are advantageous because, unlike electrochemical sensors, they do not require direct contact with the biological samples, allowing them to be reusable, reliable and not vulnerable to performance degradation with service time [14].

Validation of RF sensors can be done using either human tissue phantoms or direct testing using human subjects. Due to the difficulties of obtaining real blood samples, having additional variables such as movements, respiration, temperature variations, for preliminary testing, simulations and testing using phantom models of either animal or liquid phantoms, are preferred [15]. Animal models involving hamsters have been used in previous studies to study correlations between blood glucose and permittivity at frequencies of 10 kHz [16]. In that study, glucose solutions or various concentrations were fed to the hamster and sensor measurements were obtained at the hamster’s tail. Nowadays, phantom models are preferred as they are a more humane, cruelty-free method in which synthetic materials are used to mimic human tissues or blood vessels instead of using animals. Human tissue phantoms can also be made by mixing a combination of solutions and gels that produces the same electrical properties as human tissue [17]. The recipe for this combination of deionized water, gelatin, salt, oil, and detergent can be varied and tuned such that this mixture has the same permittivity and conductivity of human tissue at the desired frequency. Cespedes et al., in his research, tuned his mixture for applications to 5.8 GHz [17], while Yilmaz et al. designed a mixture for broadband applications of 0.3 to 20 GHz [18]. More accurate patient-specific phantom models can also be developed using synthetic anatomical models with vascular phantoms that are either 3D printed [19] or fabricated using soft lithography. In this work, we have fabricated microfluidic vascular phantoms in Polydimethylsiloxane (PDMS), which are based on angiogram images of the hand artery to validate the functionality of our sensor with different glucose concentrations.

In this work, we have presented the use of an RF sensor to monitor the different levels of blood glucose concentration. Compared to sensors with different mechanisms, RF sensors are advantageous in terms of their fast response. RF-based sensors enable sensitive differentiation of dielectric properties of the material under test that is seen as a difference in magnitude and frequency shift of S-parameters at resonance frequencies. This paper presents the design, fabrication, and testing of a stepped impedance resonator sensor for continuous in vivo blood glucose monitoring. Section 2 details the design and fabrication of the RF sensor which was fabricated using printed circuit fabrication techniques. Next, the design and fabrication of the vascular phantom using PDMS are described. The vascular phantom consists of two layers, one thin layer to mimic the permittivity of the skin layer and the second layer which contains microfluidic channels that have the design and dimensions of a hand artery. To validate the functionality of the sensor, this vascular phantom is placed underneath the RF sensor, and different glucose concentrations are injected into the channels. The scattering parameters of the sensor are recorded using a network analyzer. Section 3 presents the experimental results obtained, together with the interpretation and discussion of the results. Finally, the main conclusions inferred from this study are gathered in Section 4.

## 2. Design and Method

### 2.1. Stepped Impedance Resonator Sensor

The IDT structure is a coplanar capacitor equivalent to the parallel plate capacitor where the electrodes are placed on the same plane to provide a one-sided sensing area for any MUT. These coplanar capacitors can detect changes in permittivity of the material underneath the electrodes. If liquid is placed underneath these electrodes, any changes in the permittivity of the liquid will result in a shift of the resonant frequency of the IDT sensor. In this way, the RF sensor can detect different liquids with different values of permittivity by monitoring the changes in capacitance. The IDT sensor variables are width (*w*), length (*l*), and gap (*d*) between the electrode’s digits. Changes in width and gap of the IDT structure affect the capacitance of the sensor and allow the electrical penetration depth of the sensor to be adjusted [20] in accordance with the thickness of the material being tested.

In this work, the IDT sensor was designed to perform detection of glucose concentrations by applying frequencies in the range of 1 to 3 GHz, which are within the range of frequencies that are sensitive to changes in permittivity values of blood plasma [3]. The IDT sensor design, simulation was done using CST Microwave Studio and optimization details have been elaborated in [11]. The finalized design has the following parameters: *w* = 0.7 mm, *l* = 14 mm, *d* = 0.5 mm and number of electrodes = 20.

An important parameter that affects the sensitivity of the sensor is its Quality (Q) factor. The Q factor describes the relation between stored energy and energy usage rate and is used to describe the efficiency of the device. The basic Q-factor equation is dependent on the energy loss of the components in the device such as inductor, capacitor, or resistor. Q can also be calculated using the following equation from the S_11_ frequency response [21]

(1) $$Q=\frac{f}{\Delta f_{3dB}}=2\pi \;\frac{Energy\;stored}{Energy\;loss}=\frac{1}{R}\;\surd \frac{L}{C}$$

where f is the frequency at minimum loss, and Δ*f*_3_*_dB_* is the difference in frequencies at 3 dB drop from the maximum magnitude. This implies that, as the energy losses increase, or if there are losses in the amplitude of the signal, the *R* of the device is increased, resulting in a reduced Q factor.

**Figure 1:**
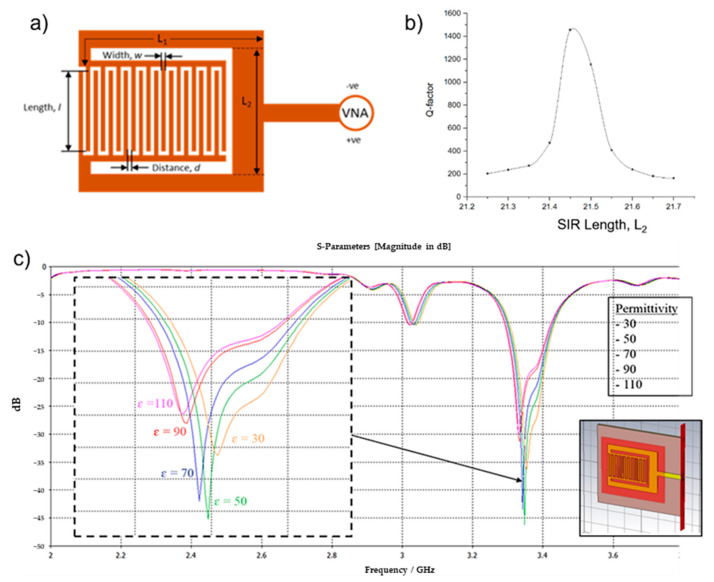
(**a**) RF Sensor comprised of interdigital and Stepped Impedance Resonator (SIR) structures and its design variables. (**b**) Quality (Q) factor values with varying SIR (L_2_) lengths. IDT with SIR design and parameter variable (**c**) Simulated frequency response of RF sensor with varying (30 to 110) blood glucose permittivity. Inset: RF sensor model in CST Microwave Studio.

The IDT sensor produced an initial Q of 200 from the simulations. To improve the Q factor, a Stepped Impedance Resonator (SIR) was added to the IDT sensor design. SIR structures are compact and have been used in filters, oscillators and mixers to improve their Q factors [22]. For our device, the SIR consists of two transmission lines of different lengths (*L*_1_ and *L*_2_) with characteristic impedances that are low impedance (*Z*_1_) and high impedance (*Z*_2_), respectively. The design parameters of both the IDT sensor and SIR are shown in Figure 1a. The addition of these two transmission lines (*L*_1_ and *L*_2_) allows us to control the capacitance of the sensor and tune the sensor to produce high Q via better impedance matching. The capacitive Equation (2) was simplified by Chomtong, P. et al. [18], as follows:

(2) $$C_{i}=\frac{\left(\varepsilon_{r}+1\right)}{L_{T}}L_{2}\left(\varepsilon_{r}+1\right)\left[0.1\left(n-3\right)+0.11\right]$$

where $C_{i}$ is the capacitive value of the IDT, $L_{T}$ is the total length of the IDT, $L_{2}$ is the length of SIR, n is the number of fingers and $\varepsilon_{r}$ is relative permittivity. The design parameter of SIR is controlled by both length and impedance ratio.

To optimize the Q factor, simulations were done using CST Microwave Studio in which the SIR length was varied, and the sensor’s frequency response was obtained. The design dimension unit was set in millimeters (mm). The simulations were performed in a frequency range of 1 GHz to 5 GHz. Based on the above equation, L_1_ has no significant effect on the performance of SIR and was kept constant for the simulations, while L_2_ varied between 21.2 and 21.7 with a step of 0.1. The Q factor of each L_2_ value was calculated based on the RF sensor’s simulated frequency response, and the plot of Q versus L_2_ is shown in Figure 1b. The highest value of Q factor obtained was 1455 when L_2_ = 21.45 mm. Q factor values of near or over 1000 at frequencies between 100 MHz and 1 GHz are suitable for sensing applications and can adequately reduce high-frequency losses [23].

To evaluate the performance of the device for sensing applications, another simulation was done in which the dielectric values near to the sensor’s surface were changed to have blood permittivity values. A new material block was placed underneath the sensor design (Figure 1c (inset)) and its permittivity values were varied from 30 to 110 with increments of 20. This method allows us to observe the change in frequency response of the sensor due to the varying of blood glucose levels. Details on the boundary conditions of the sensor can be found in [11]. The sensor’s S_11_ frequency response was plotted and is shown in Figure 1c. All values used for blood in the CST simulations are listed in Table 1. From the simulations, higher permittivity values result in a decrease in resonant frequencies. In terms of glucose concentrations, lower glucose concentrations also correspond to higher permittivity values.

Based on the simulation results, three different sensor designs were selected for fabrication: Sensor A (SIR width = 21.40 mm), Sensor B (SIR width = 21.45 mm), Sensor C (SIR width = 21.50 mm). The sensors were fabricated on a Flame-Retardant 4 (FR4) substrate, a common substrate for printed circuits. The FR4 printed circuit board has a thickness of 1.57 mm, with relative permittivity of 4.7, and loss tangent of 0.014. The electrodes are from copper due to its high electrical conductivity. The sensor design was first drawn in Adobe Illustrator and printed on a transparency to form a positive mask. The design is then fabricated onto the FR4 substrate using the same techniques as the printed circuit process which involves exposure, development, etching, and finally stripping. Once the circuit board is ready, it is tested with a multimeter to ensure there is no short and a female SMA connector is attached as the input and output port of the sensor.

### 2.2. Microfluidic Vascular Phantom

Patient-specific vascular models have been used for surgical training to simulate complex procedures such as endovascular aneurysm repair and coil embolization [19]. In this work, vascular model of the ulnar artery of humans was translated into a microfluidic device using angiogram images of the hand artery. Creating a microfluidic phantom specifically to test the RF sensor instead of human subjects allows us better control and also simplifies the experiments. Using this method, multiple glucose concentrations are injected into the channel repeatedly without harming the subjects. Other environmental factors such as temperature, and flow rate of the liquid being tested, can also be made constant during experimentation.

**Figure 2:**
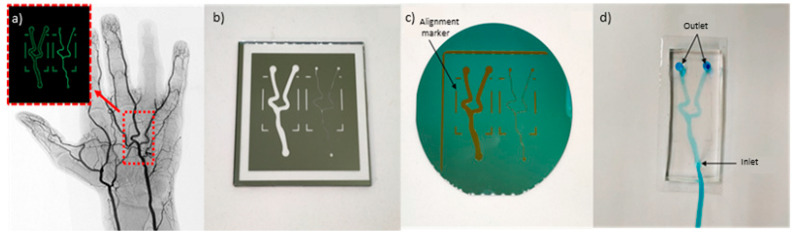
(**a**) Angiogram image of a hand artery from [19]. Inset: Replica image of hand artery redrawn in Adobe Illustrator. (**b**) Design fabricated on chromium on glass mask. (**c**) Microfluidic pattern on dry photoresistant film fabricated using soft lithography. (**d**) Microfluidic device fabricated using PDMS.

Dimensions of the ulnar artery were obtained from Fazan et al. [24], who studied 46 male, embalmed human cadavers. In this study, the mean diameter of the ulnar artery on the right hand was found to be approximately 2.5 ± 0.2 mm. The design of the channels was drawn in Adobe Illustrator, as shown in Figure 2a, to closely mimic the angiogram images of the hand artery obtained from [25]. Angiography typically involves injection of a radio-opaque contrast agent into the blood vessels and, using the X-ray imaging, the blood vessel shapes can be captured. The design was then fabricated into a chromium on glass mask, as shown in Figure 2b. Soft lithography methods were used to produce the design mold, as shown in Figure 2c. Fabrication steps are detailed in Section 2.3. The fabricated microfluidic device in PDMS is shown in Figure 2d. The size of the microfluidic device corresponds to the size of the RF sensor, which is 40 × 50 mm, and alignment markers were included in the mask design to provide constant placement of the channel on the sensor when taking the experimental measurements.

**Figure 3:**
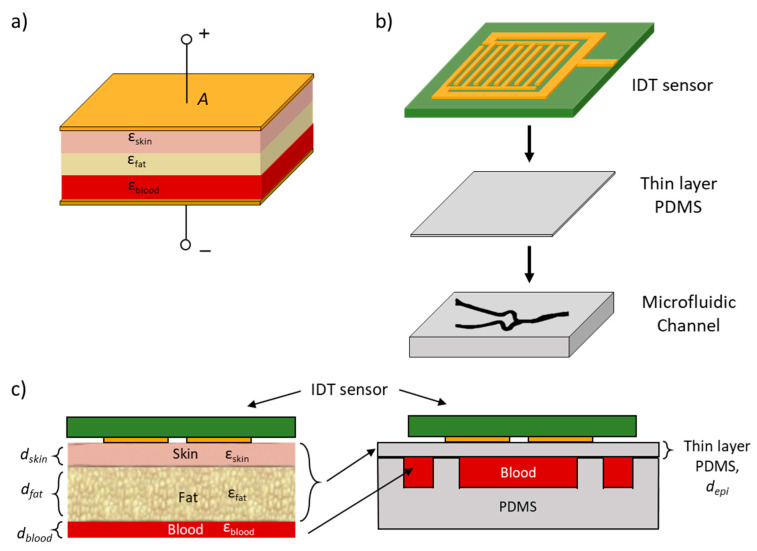
**(a)** Dielectric stack model of skin, fat and blood between two capacitive plates. (**b**) Layer stack of RF sensor on thin PDMS and microfluidic channel used for testing. (**c**) Left: Layer stack of RF sensor on human hand comprising of three layers: skin, fat and blood. Right: Microfluidic vascular phantom layer stack that was used for testing where the thickness of the thin-layer PDMS was designed such that it has the same permittivity values of the three layers (skin, fat and blood).

When the sensor is placed on a human hand, the capacitive model of the layers can be represented as a 3-layer stack of skin, fat and blood, as shown in Figure 3a. The interdigital sensor is represented as capacitive parallel plates. To accurately model the dielectrics that are present in the hand, a thin layer of PDMS is placed in between the sensor and the microfluidic channel, as shown in Figure 3b. The dielectric value of this thin PDMS layer should match the dielectric values of skin and fat, while the dielectric value of blood is the unknown. The total capacitance of skin and fat can be expressed as follows:

(3) $$\frac{1}{C_{T}}=\frac{1}{C_{skin}}+\frac{1}{C_{fat}}$$

(4) $$C_{T}=\varepsilon_{0}A\frac{\varepsilon_{skin}\varepsilon_{fat}}{\varepsilon_{fat}d+\varepsilon_{skin}d_{fat}}$$

where *C_T_* is total capacitance, *C_skin_* and *C_fat_* is the capacitance, *ε_skin_* and *ε_fat_* is the dielectric constant, and *d_skin_* and *d_fat_* is the average thickness for skin and fat respectively. The expression (4) was derived from (3). The total capacitance, *C_T_*_,_ can then be used to find the thickness of the thin-layer PDMS, as follows:

(5) $$d_{\mathrm{PDMS}}=\frac{\varepsilon_{\mathrm{PDMS}}\left(\varepsilon_{fat}d_{skin}+\varepsilon_{skin}d_{fat}\right)}{\varepsilon_{skin}\varepsilon_{fat}}$$

where ε_PDMS_ is the dielectric constant of PDMS. Substituting the parameter values in Table 2 [26] into Equation (5), the thickness of the bottom layer PDMS was theoretically calculated to be 900 µm. The planar scheme of the RF sensor on a human hand is shown in Figure 3c (left), and the cross-section of the microfluidic vascular phantom is shown in Figure 3c (right).

### 2.3. Microfluidic Fabrication

**Figure 4:**
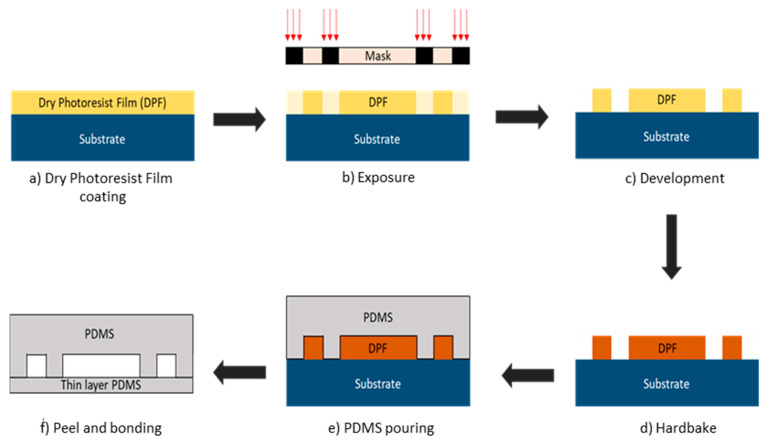
Microfluidic soft lithography fabrication steps. (**a**) Silicon wafer substrate is laminated with dry photoresist film (DPF). (**b**) Microfluidic pattern from mask was exposed with UV light and transferred to DPF. (**c**) Substrate with DPF was immersed in developer solution to remove the layer that is exposed to the UV light. (**d**) The sample was baked to harden the DPF. (**e**) PDMS was poured on to the design mold. (**f**) PDMS was peeled off from the mold and bonded with the thin layer PDMS.

The microfluidic blood vessel pattern was developed into a chromium photomask (Delta Mask B.V., The Netherlands) for soft lithography. The final product is shown in Figure 2b. For soft lithography, a silicon wafer is laminated with dry photoresist film at 100 °C using a laminator (RSL-382S) and covered with an adhesion promoter. The microfluidic pattern from the mask was then transferred onto the dry photoresist film using a Suss MicroTec mask aligner MJB4 [27] via photolithography to form the microfluidic mold. After exposure, the sample is heated on a standard hotplate at 80 °C for 2 h. Next, the sample was immersed in a developer solution (cyclohexanone) which dissolves areas of the dry film that were exposed to light. The sample is then baked in an oven or hot plate at temperatures between 100 and 120 °C. This is needed to drive off liquids that may have been absorbed on the substrate and to crosslink the remaining photoresist layer of the mold. Once hardened, this mold can be peeled off from the silicon substrate. These steps are illustrated in Figure 4a–e.

As shown in Figure 4f, there are two PDMS layers, one thin to represent the human skin and fat, and another thicker layer with microfluidic channels that represents the blood vessels. To prepare the PDMS layers, Sylgard 184 silicone elastomer with elastomer curing agent (a crosslinking agent) is mixed at a 10:1 ratio in the plastic cup. Air bubbles were removed from the PDMS mixture by degassing it for 1 h. This mixture can then be used to form both layers: thin-layer PDMS and microfluidic channels. To produce the thin layer, the liquid PDMS was spin coated in a spin coater (Karl SUSS RC8) at a speed of 2500 rpm for 30 s to obtain a layer thickness of around 900 μm. Once the layer is formed, it is baked in an oven at 100 °C for 30 min to solidify the PDMS mixture. The solid PDMS layer was then peeled off and cut into the desired shape using a razor blade.

For the fabrication of microfluidic channels, the liquid PDMS is poured onto the microstructure mold and heated in an oven at 85 °C for 15 min to obtain a hardened, elastomeric replica of the mold. The hardened PDMS layer was then peeled off and cut using a razor blade into a rectangular shape. Two circular holes were punched into the PDMS layer near the two ends of the capillary channel to make the holes for the inlet and outlet media reservoirs. Both layers of PDMS (thin layer and microfluidic layer) were treated with oxygen plasma before assembly. Oxygen plasma treatment promotes adhesion between the two layers and allows very strong bonding that avoids any leakage. The two PDMS layers were then assembled, and a polyethylene tube (length = 900 mm and inner diameter = 840 µm) was connected at the inlet of the microfluidic channel. The tube can be connected later to a disposable syringe and a syringe pump for testing.

**Figure 5:**
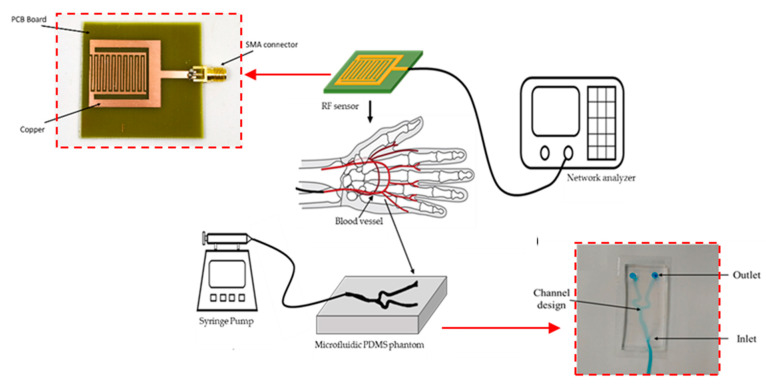
Experimental setup and placement of RF sensor on top of the vascular phantom. A syringe pump was used to inject fluids with different glucose concentrations to mimic the flow of blood with varied sugar levels. (Inset: Fabricated RF Sensor and fabricated microfluidic vascular phantom).

Figure 5 shows the fabricated sensor (a), the testing scheme using the microfluidic vascular phantom in lieu of a human hand (b). The microfluidic device was connected to a syringe pump. Measurements of glucose levels using the RF sensor is done without direct contact with blood, allowing the sensor to be reusable as there is no contamination. The fabricated sensor is connected to a network analyzer and the frequency response at each concentration can be obtained. The usage of a microfluidic vascular phantom eases the testing methods and reduces the need of using human subjects at the prototype level.

### 2.4. Glucose Sample Preparation

D(+)-Glucose anhydrous [C_6_H_12_O_6_] was used to prepare the glucose concentration. The sample glucose (180.16 g/mol) was diluted with DI water at normal room temperature. Glucose solution with concentrations ranging from 30 mg/dL to 240 mg/dL with an increment of 30 mg/dL was prepared to match with the different blood sugar levels (hyperglycemic, normal, hypoglycemic) that may exist in diabetic patients, as shown in Table 3 [28].

### 2.5. Experimental Setup

**Figure 6:**
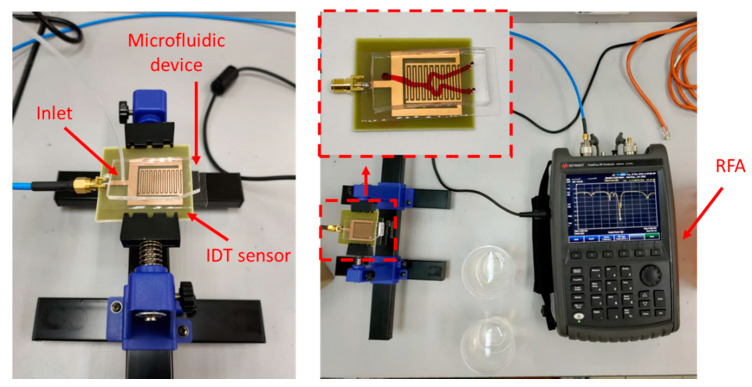
Experimental setup used for RF sensor measurements. Left: The microfluidic device is placed on top of the RF sensor. The inlet of the microfluidic channel is connected to a syringe pump. Right: The RF sensor is connected to a Keysight Fieldfox RF Analyzer.

Measurement of RF sensor frequency response was made using Keysight Fieldfox RF Analyzer (RFA). Figure 6 shows the placement of the microfluidic device on top of the RF sensor. A male SMA cable was connected to the sensor and the RFA to measure the S_11_ parameters of the sensor. A disposable syringe that contains the appropriate glucose concentration was placed in the syringe pump and the tube was connected to the inlet of the microfluidic device.

The RFA was calibrated with a Short, Open, Load, Through (SOLT) SMA calibration kit prior to measurements. The calibration was carried out by measuring a SOLT termination at the point where the sensor will be measured, which is directly at the port. The calibration and measurement frequencies of the RFA were set between 1 GHz and 5 GHz. All three different sensor designs were measured with two different microfluidic devices. The two microfluidic devices only differ in terms of the materials used as their substrate namely: thin-layer PDMS and glass slide. Experimental measurements were done in triplicate where measurement of S_11_ values for each glucose concentration was repeated three times (*n* = 3). The RF sensor was aligned with the fluidic device using the alignment markers to ensure constant placement for each experiment to ensure precision and repeatability of the measurements. The measurements were performed in triplicate for each sensor with each type of microfluidic device to ensure the conformities of the readings.

To imitate the blood fluid properties in human arteries, the flow rate inside the channel must be uniform and constant throughout the measurement. To achieve this requirement, a syringe pump was used separately at the inlet of the microfluidic device. The flow of the pump was initially set at 0.5 mL/min and slowly increased until the speed reached an optimal rate. Air bubbles were formed and trapped in the channel at the junction of the artery model at a flow rate of 0.5 mL/min. This indicates that the pressure of the liquid injection is quite low, thus prone to bubble formation inside the channel. Bubbles fully disappeared once the rate was increased to 3.2 mL/min. This rate also falls inside the range of normal blood flow rate in human arteries, which is 3.0–26 mL/min, as discussed in [29], and was used throughout the experiments. Further increase in the flow rate could result in leakage within the channels.

## 3. Results and Discussion

Three different sensor designs were measured and produced different quality factors, namely: Sensor A (SIR width = 21.40 mm, Q factor = 980), Sensor B (SIR width = 21.45 mm, Q factor = 1455), Sensor C (SIR width = 21.50 mm, Q factor = 1154). Next, measurements with the microfluidic devices were conducted as shown in Figure 6. RF sensors are advantageous as the response time of the RF sensors only depends on the sweep period of the vector network analyzer used for taking measurements [14]. Vector network analyzers have been reported to have short detection times: 0.85 s in [14] and less than 2 s in [13].

**Figure 7:**
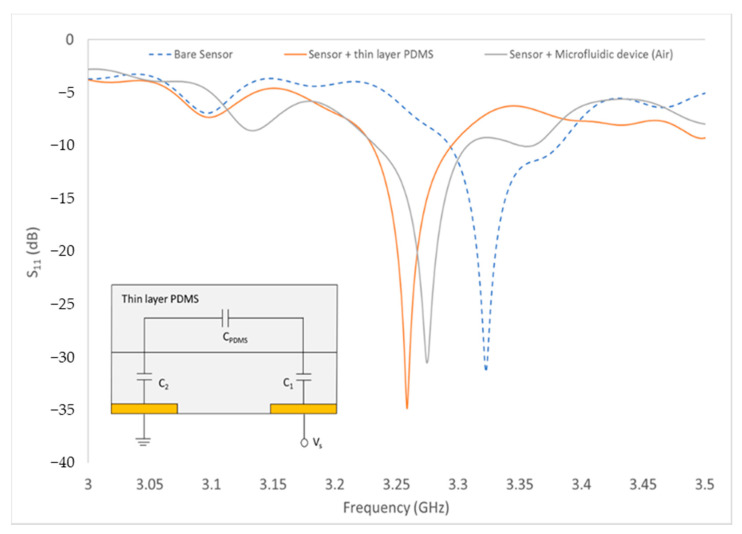
Measurement of the bare sensor, sensor with thin-layer PDMS and sensor with microfluidic device. (Inset: Equivalent circuit of RF sensor with thin-layer PDMS).

Only measurements of Sensor B with the thin PDMS as its substrate are shown as it produced the highest quality factor. The S_11_ frequency response for RF sensor B was plotted as shown in Figure 7 and the values of frequency (*f* = 3.2548 GHz), magnitude (S_11_ = −34.873 dB) and phase angle (*θ* = −155.487) were used to find the capacitance value of thin-layer PDMS. The load impedance formula shown in (6) was used to convert S_11_ to capacitance value.

(6) $$Z_{L}=Z_{0}\left(\frac{1+S_{11}}{1-S_{11}}\right)$$

where *Z_L_* is load impedance and *Z*_0_ = 50 ohms. From the results in *Z_L_*, the imaginary part is the series reactance and negative values indicate that it is capacitive. From calculations, *Z_L_* = 48.38−0.723*i* and the reactance value was converted to find the capacitance of the thin-layer PDMS. The capacitance value of thin-layer PDMS (*C*_PDMS_ = 6.756 × 10^−11^ F) was used to find the relative permittivity of the PDMS, ε_PDMS_. The measured ε_PDMS_ was found to be 0.389.

**Figure 8:**
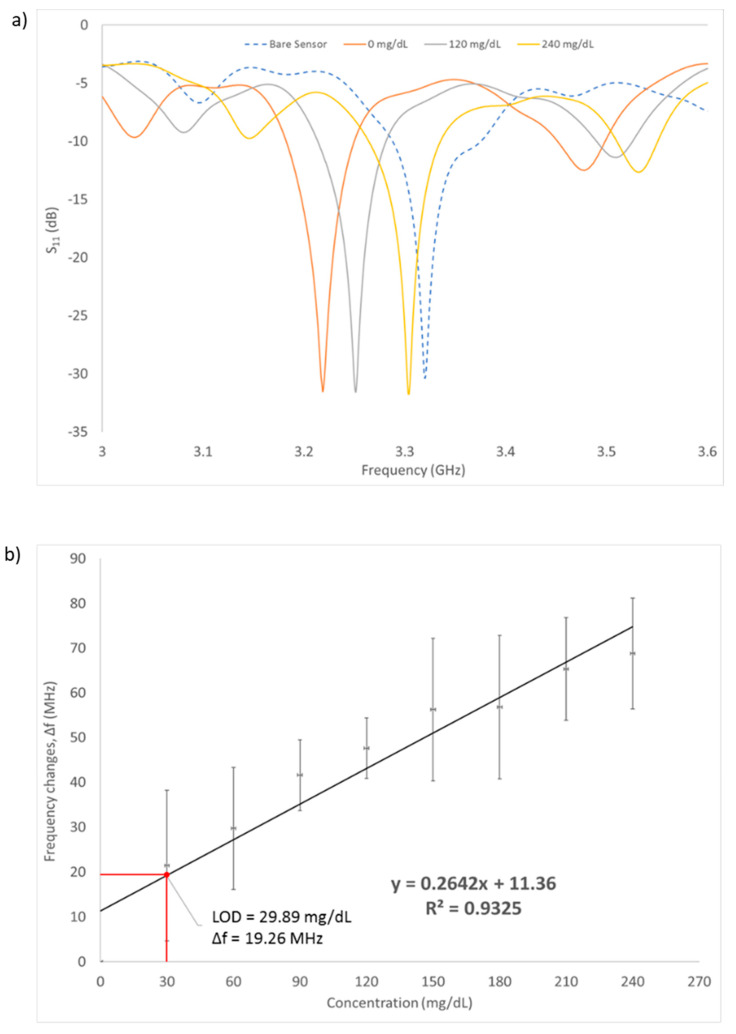
(**a**) S_11_ measurement results of the sensor with varying glucose concentration (0, 120, 240 mg/dL). (**b**) Frequency changes with increasing value of glucose concentration. Limit of detection was found to be 29.89 mg/dL. Measurements were made in triplicates. Error bars are also shown in the graph.

Measurement of the microwave reflection coefficient S_11_ was done on a series of aqueous glucose concentrations flowing inside the microfluidic channel. The glucose concentrations ranged from 0 mg/dL (DI water) to 240 mg/dL, which is in accordance with the human blood glucose levels. Figure 8a shows the pattern of resonance frequency shifting towards the different glucose concentrations (0, 120, 240 mg/dL). As can be seen, this graph shows the relationship between the frequency change response against the sample glucose concentration. As the glucose concentrations are increased, the frequency is also increased, shifting the resonance frequency of the S_11_ measurement to the right side after each concentration changed. The RF sensor can detect different liquids with different values of permittivity. The difference in permittivity value will cause changes in the resonance frequency shift.

A linear regression fitted line was plotted on the graph and the correlation coefficient R-square = 0.9325. The regression line indicates that the change of frequency is directly proportional to the concentration changes of the glucose. This suggested that different glucose concentrations have different dielectric permittivity properties, which can be detected by measuring the change in capacitance, and therefore, change in resonance frequency (S_11_) of the RF sensor. This is in accordance with the Cole–Cole model theory [30] where the variation of reflection coefficient S_11_ leads to the frequency shift over the glucose concentration. Increasing the glucose concentrations in a liquid would generally decrease the permittivity of the liquid over the frequency; thus, in turn shifting the resonance frequency to a higher value.

The graph shows a gradually increasing pattern as the number of concentrations is increased and the trendline from the curve was added to find the sensitivity (slope) and the limit of detection (LOD). Based on the linear regression equation shown in Figure 8b, the detection sensitivity of the biosensor is 264.2 KHz/mg·dL^−1^. The LOD of the sensor was calculated to be at 29.89 mg/dL using the formula of 3σ/m, where σ is the standard deviation of blank solution and m is the slope of the graph. This implies that the sensor can be used to detect glucose changes greater than approximately 30 mg/dL. The sensor has successfully detected the three human glucose levels: below 60 mg/dL (hypoglycemic), 72–108 mg/dL (normal) and above 200 mg/dL (hyperglycemic). To achieve the optimal sensing performance of the sensor, the regression coefficient needs to be improved to the value of 0.99. This can be done by fixing the location of the cables when setting up the experiment such that it remains constant throughout all the measurements. This will result in improvements in the sensitivity and LOD of the sensor. As a comparison with existing research, Table 4 shows the performance of other types of remote sensors in terms of their LOD, frequency of operation and range of glucose level detection.

The advantage of this work is that other than the high-Q sensor design, measurements were conducted with a vascular phantom, which is a closer mimic to blood vessels compared to tissue phantoms which were used in other works. The skin and fat dielectric permittivity values were also represented as a single, thin PDMS layer to improve similarity to the human model. In addition, the RF sensor has a measured LOD of 29.89 mg/dL, which is lower compared to the other works. These results show that this biosensor has the potential of being applied as a low-cost non-invasive glucose sensing.

## 4. Conclusions

In this paper, an interdigital electrode with SIR structures was used for remote sensing of different glucose concentrations. The sensor was fabricated on a double-sided PCB using a conventional PCB manufacturing process. A microfluidic device was also fabricated to imitate the real blood vessel structure of the human hand and form a microfluidic vascular phantom to ease testing. The optimal flow rate to flow glucose inside the channel was found to be at 3.2 mL/min where all bubbles completely disappeared from the channel. A wide range of glucose concentrations (0 mg/dL to 240 mg/dL) was used to test the sensor’s performance. The reflection coefficient, S_11_ and the resonance frequency are sensitive to the glucose changes inside the microfluidic hand vascular model. The sensor showed a linearly proportional relationship between resonance frequency changes and varying glucose concentrations. The sensitivity of the sensor was found to be 264.2 kHz/mg·dL^−1^ with LOD of 29.89 mg/dL. This sensor has the potential of being used as a device for continuous, remote monitoring of blood sugar levels for diabetic and prediabetic patients. Non-invasive, continuous monitoring of sugar levels for vulnerable individuals allows early interventions and results in better quality of life.

## Figures and Tables

**Table 1:** Important blood parameter values used in the simulation.

Parameter	Values
Electric Conductivity (S/m)	3.05
Density (kg/m^3^)	1050
Heat Capacity (J/kg/°C)	3617
Thermal Cond. (W/m/°C)	0.52
Heat Transfer Rate (mL/min/kg)	10,000

**Table 2:** Specific parameter values for skin and fat of human body on ulnar artery.

Parameter	Dielectric Constant, ε	Thickness [mm]
Skin	37.50	1.5
Fat	10.70	3.0
Blood vessel	41.90	2.5
PDMS	2.68	0.9

**Table 3:** Range of blood glucose concentrations for different sugar levels in the blood [28].

Blood Sugar Levels	Blood Glucose Concentration (mg/dL)
Hyperglycemia	>200
Normal Glycemia	72–108
Hypoglycemia	<60

**Table 4:** The value of specific parameters of skin and fat of human body on ulnar artery.

Reference	Sensor	Phantom	Frequency	LOD	Range Glucose Level
[13]	Dual needle	Hamster tail	10 kHz	-	20–500 mg/dL
[14]	Dielectric probe	Oil, gelatin, salt, deionized water, detergent	4–7 GHz	100 mg/dL	0–400 mg/dL
[15]	Dielectric probe	Oil, gelatin, salt, deionized water, detergent	0.3–20 GHz	72 mg/dL	72–216 mg/dL
This work	Stepped impedance resonator	Dual-layer PDMS	1–5 GHz	29.89 mg/dL	0–240 mg/dL

## Data Availability

The study did not report any data.
